# Comprehensive dissection of transcript and metabolite shifts during seed germination and post-germination stages in poplar

**DOI:** 10.1186/s12870-019-1862-3

**Published:** 2019-06-26

**Authors:** Chunpu Qu, Zhuang Zuo, Lina Cao, Jiahuan Huang, Xue Sun, Peng Zhang, Chengjun Yang, Lixin Li, Zhiru Xu, Guanjun Liu

**Affiliations:** 1 State Key Laboratory of Tree Genetics and Breeding (Northeast Forestry University), School of Forestry, Northeast Forestry University, Harbin, 150040 People’s Republic of China; 2 School of Forestry, Northeast Forestry University, Harbin, 150040 People’s Republic of China; 3 College of Life Science, Northeast Forestry University, Harbin, 150040 People’s Republic of China; 4 Key Laboratory of Saline-Alkali Vegetation Ecology Restoration, Ministry of Education, Alkali Soil Natural Environmental Science Center, Northeast Forestry University, Harbin, 150040 People’s Republic of China

**Keywords:** Poplar, Seed germination and post-germination stages, Transcriptomics, Metabolomics, Data integration, Candidate genes

## Abstract

**Background:**

Seed germination, a complex, physiological–morphogenetic process, is a critical stage in the life cycle of plants. Biological changes in germinating seeds have not been investigated in poplar, a model woody plant.

**Results:**

In this study, we exploited next-generation sequencing and metabolomics analysis and uncovered a series of significantly different genes and metabolites at various stages of seed germination and post germination. The *K*-means method was used to identify multiple transcription factors, including *AP2/EREBP*, *DOF*, and *YABBY*, involved in specific seed germination and post-germination stages. A weighted gene coexpression network analysis revealed that cell wall, amino acid metabolism, and transport-related pathways were significantly enriched during stages 3 and 5, with no significant enrichment observed in primary metabolic processes such as glycolysis and the tricarboxylic acid cycle. A metabolomics analysis detected significant changes in intermediate metabolites in these primary metabolic processes, while a targeted correlation network analysis identified the gene family members most relevant to these changing metabolites.

**Conclusions:**

Taken together, our results provide important insights into the molecular networks underlying poplar seed germination and post-germination processes. The targeted correlation network analysis approach developed in this study can be applied to search for key candidate genes in specific biochemical reactions and represents a new strategy for joint multiomics analyses.

**Electronic supplementary material:**

The online version of this article (10.1186/s12870-019-1862-3) contains supplementary material, which is available to authorized users.

## Background

Plant seed germination and its immediate aftermath is a very complicated biological process involving a series of morphological, physiological, and biochemical changes under environmental influence [1–5]. Extensive research at both physiological and morphological levels has been performed on germination and post-germination processes of herbaceous plants such as Arabidopsis [6–9], soybean [10–12], rice [13–17], and barley [18–20]. In recent years, an ‘omics’ approach has been used to obtain information on changes in the levels of an entire range of metabolites, proteins, and gene transcripts [21–28]. The results of such studies provide an integrated view of the biological process of seed germination and post-germination events.

Poplar is a model woody plant that has long been studied, including in terms of physiological and morphological changes during germination [29]. Previous research on rapid asexual propagation and tissue culture of poplar has been highly successful [30, 31]. In comparison, few studies have been carried out on molecular mechanisms of poplar seed germination (sexual reproduction), although several preliminary reports on the relationship between seed germination and environment have recently appeared [28, 32–34]. Investigation of the molecular mechanism of poplar seed germination and its immediate aftermath may be key to the elucidation of this process in woody plants.

By definition, seed germination begins with the uptake of water by the mature dry seed and terminates with the protrusion of the radicle through the seed envelope. On the basis of changes in fresh weight during seed germination, a three-phase germination model has been widely adopted in previous studies. The three phases of this model are as follows: I, rapid imbibition of water, which occurs as long as all matrices and cell contents are fully hydrated; II, limited water uptake; and III, increasing water uptake accompanied by embryo axis elongation and breakthrough of covering layers to complete germination (visible germination) [1, 35, 36]. Seeds subsequently enter a post-germination stage until expansion of the first true leaf. In the present study, we used these three phases as a foundation and divided the poplar seed germination process into six stages ranging from stage 2, rapid imbibition of water, to stage 6, expansion of the first true leaf, with seeds before imbibition defined as stage 1. Using next-generation sequencing and high-performance liquid chromatography-mass spectrometry (HPLC-MS/MS), we generated transcriptome and metabolome data for six seed-germination stages in poplar. We then applied *K*-means clustering and weighted gene coexpression network analysis (WGCNA) to identify stage-specific gene clusters and network modules. In addition, we found that genes involved in cells and stress response were enriched at certain stages, whereas primary metabolic processes such as glycolysis and the tricarboxylic acid cycle (TCA) did not change significantly. Network analysis of enriched modules at different stages revealed a series of highly connected hub genes involved in transport, cell walls, and signaling. Finally, targeted correlation network analysis (TCNA) allowed us to find the most relevant gene family members potentially related to metabolite changes. Taken together, our results lay a foundation for further biochemical and functional analysis of poplar seed germination and its aftermath.

## Results

### Global analysis of RNA-Seq data

**Fig. 1:**
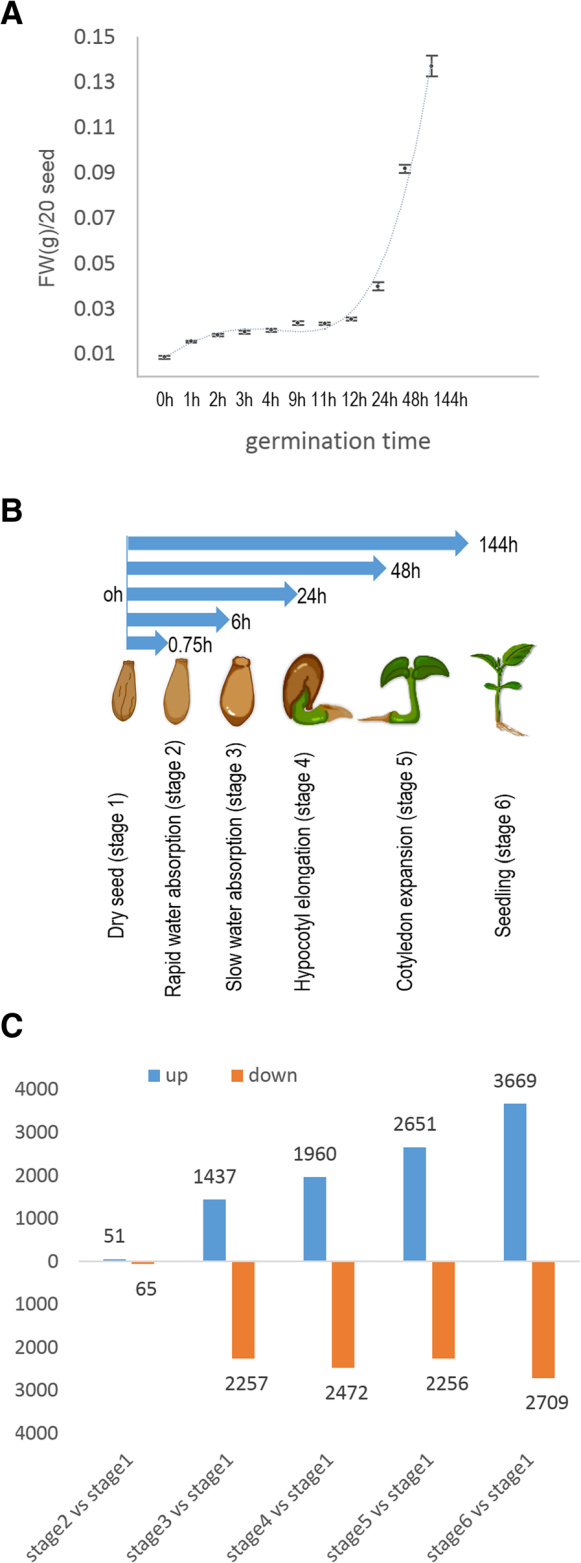
Schematic representation of poplar seed germination and the number of significantly changed transcripts between successive stages during poplar germination and post germination. **a** Changes in fresh weight after seed imbibition. **b** Schematic representation of the experimental setup. **c** Summary of the number of significantly changed transcripts between successive stages during poplar germination and post germination

The poplar seed germination process was divided into stages 2 to 4 of the seed germination stages defined by Bewley (1997) [1]. As assessed by changes in seed fresh weights after water absorption, periods of rapid and slow water absorption were defined as stages 2 and 3, respectively (Fig. 1a and Additional file 1: Table S1), while the hypocotyl extension period was defined as stage 4. We collected samples at a total of six stages: stages 2 to 4, the dry seed phase (stage 1), and post-germination stages of cotyledon unfolding (stage 5) and true-leaf unfolding (stage 6). A schematic representation of the experimental setup is shown in Fig. 1b.

For samples from each stage, the average number of raw reads per library was approximately 27 million (Additional file 2: Table S2), with 72% of these raw reads mapped to the poplar reference genome. Normalized read counts were calculated as FPKM values (expected number of fragments per kilobase of transcript sequence per millions of base pairs sequenced). A total of 63,498 genes, which corresponds to approximately 90% of the 73,000 genes predicted in the poplar genome, were found to be expressed during seed germination and post-germination stages. Based on the results of differential expression analysis using NOISeq, up- and down-regulated genes were identified for each pairwise comparison according to the criteria of fold change ≥2 and *p* ≥ 0.8. The number of observed transcript changes was as follows: 116 between stages 1 and 2 (51 up, 65 down), 3694 between stages 1 and 3 (1437 up, 2257 down), 4432 between stages 1 and 4 (1960 up, 2472 down), 5207 between stages 1 and 5 (2651 up, 2256 down), and 6378 between stages 1 and 6 (3669 up, 2709 down). Compared with other stages, fewer changes in the number of transcripts were observed between stages 1 and 2. The number of genes with significant expression changes between successive time points during poplar germination and post germination is shown in Fig. 1c.

### Identification of temporal expression trends across poplar seed transcriptomes during germination and post germination

**Fig. 2:**
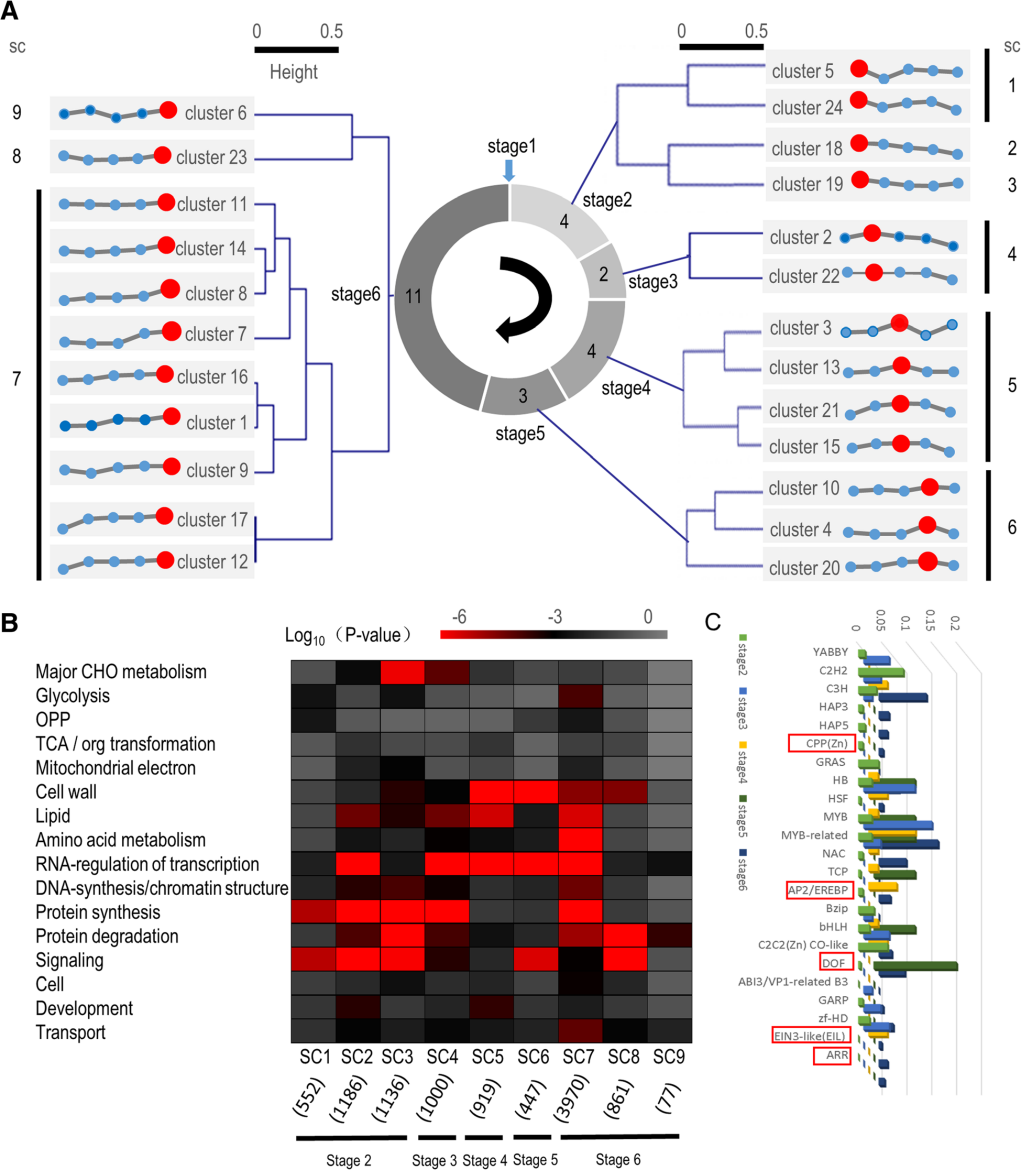
Identification of unique stage-specific expression trends by *K*-means clustering. **a** Twenty-four clusters representing 10,148 genes with distinct stage-specific expression patterns. These clusters were further grouped into nine superclusters (SC1–SC9). The red dot in each cluster is the stage with the greatest combined fold-change of genes. **b** Enriched MapMan categories for each of the nine superclusters (SC1–SC9). Significant enrichment is indicated by a low *p*-value. Numbers in parentheses represent the number of genes included in each superfamily. **c** Distribution of transcription factors in each cluster. Genes that are expressed at only one stage are boxed in red

To reveal the variation of differentially expressed genes (DEGs), *K*-means clustering was used to group and visualize RNA-Seq expression profiles of all genes differentially expressed during seed germination at each given time point, which corresponded to 10,148 genes in total. All 24 clusters are shown in Fig. 2a, and their gene IDs are listed in Additional file 3: Table S3. Each identified gene cluster could be assigned to a specific germination stage on the basis of its highest expression: clusters 5, 18, 19, and 24 to stage 2; clusters 2 and 22 to stage 3; clusters 3, 13, 15, and 21 to stage 4; clusters 4, 10, and 20 to stage 5; and clusters 1, 6–9, 11, 12, 14, 16, 17, and 23 to stage 6.

Clusters with similar expression trends were further combined into nine superclusters (Fig. 2a). To understand the distribution of genes in these superclusters, an enrichment analysis was performed with the MapMan tool to identify specific categories [37]. In supercluster 1, the top enriched MapMan category was signaling, followed by protein synthesis, glycolysis, and oxidative pentose phosphate pathway (OPP). The top enriched category in supercluster 2 was also protein synthesis, followed by signaling, RNA regulation of transcription, lipids, and protein degradation. Protein degradation was predominant in supercluster 3, followed by protein synthesis, signaling, major CHO metabolism, and DNA synthesis/chromatin structure. The top enriched categories in supercluster 4 were protein synthesis, RNA regulation of transcription, lipids, and major CHO metabolism; those in supercluster 5 were cell walls, RNA regulation of transcription, lipids, and development. In supercluster 6, the most enriched category was RNA regulation of transcription, which was followed by cell walls, signaling, and transport. Superfamily 7 had the largest number of highly enriched categories, including protein synthesis, RNA regulation of transcription, amino acid metabolism, and lipids. In supercluster 8, the top enriched MapMan category was protein degradation, followed by signaling, cell walls, and transport. Most categories in superfamily 9 were only lowly enriched, with only protein degradation and transport having relatively high enrichment. Noteworthily, two categories, TCA/org transformation and mitochondrial electron transport/ATP synthesis, were always lowly enriched. These results indicate that the number of active genes associated with energy, TCA, and substance conversion was basically constant (Fig. 2b).

Genes related to RNA regulation of transcription, mostly encoding transcription factors, were relatively highly enriched. Five types of transcription factors, *CPP (Zn)*, *AP2/EREBP*, *EIL*, *ARR*, and *DOF*, were only expressed at one stage (Fig. 2c and Additional file 4: Table S4). *YABBY* transcription factors were specifically expressed during early germination stages, and *EIN3-like (EIL)* and *ARR* were only expressed at the end of germination (Fig. 2c). These results are consistent with previous research. For example, Howell et al. (2009) found that expression levels of *AP2/EREBP* family genes increased during early stages of seed germination in rice. This family is considered to be related to water uptake and ABA signaling during early rice seed germination [25]. The dynamic and stage-specific expression patterns of these transcription factors probably reflect their key functions during different germination stages.

### Coexpression network analysis with WGCNA

**Fig. 3:**
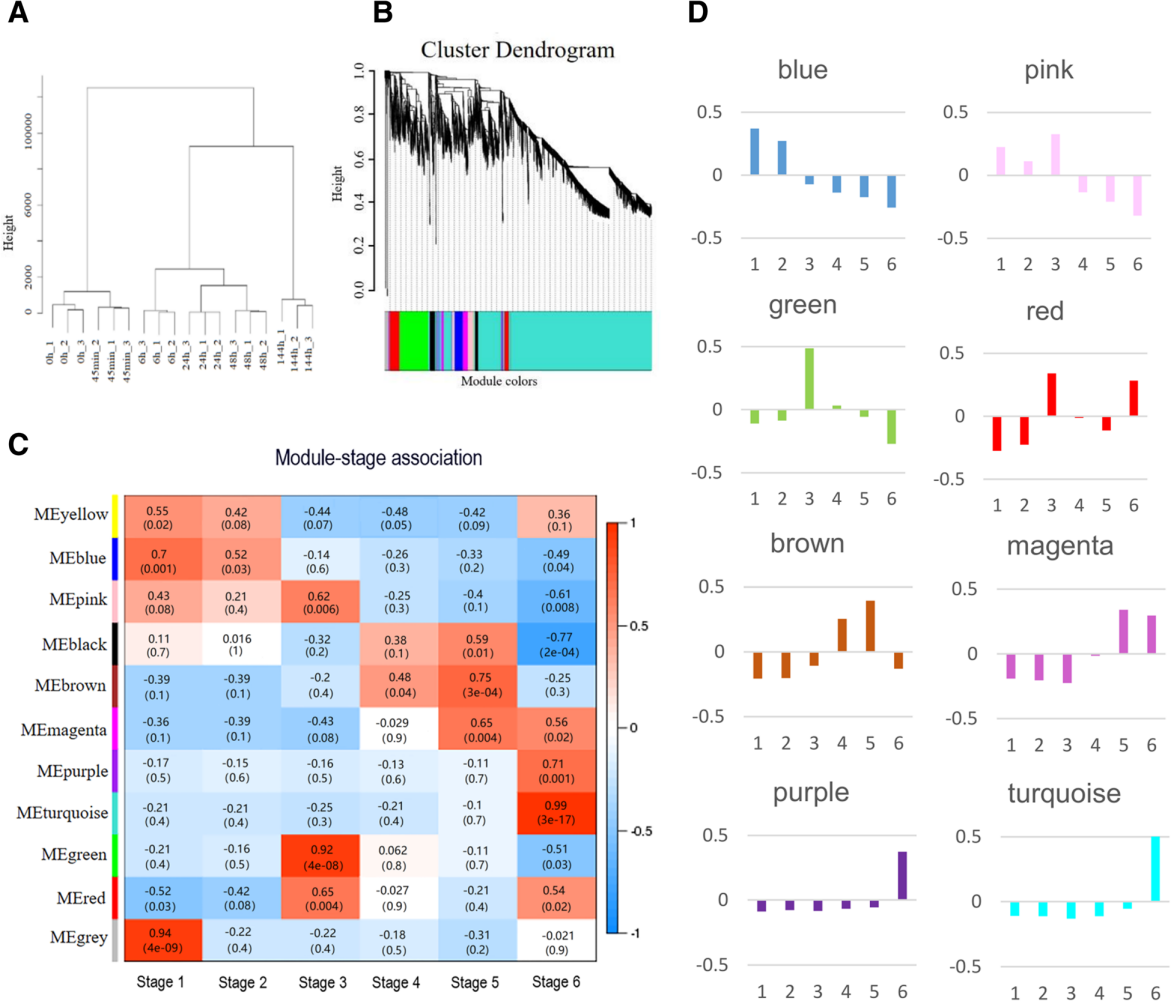
Weighted gene co-expression network analysis (WGCNA) of significantly changed transcripts. **a** Cluster dendrogram showing global relationships among different seed germination and post-germination stages. **b** Hierarchical cluster tree showing coexpression modules identified by WGCNA. Each leaf in the tree is one gene. The major tree branches constitute 11 modules labeled by different colors. **c** Module–stage association. Each row corresponds to a module, and each column represents a specific stage. The color of each cell at the row–column intersection indicates the correlation coefficient between a module and the stage. A high degree of correlation between a specific module and stage is indicated by red. **d** Eigengene expression profile of each module. The y-axis indicates the value of the module eigengene; the x-axis indicates the sampled germination/post-germination stage (1–6)

To understand the biological process of poplar seed germination and its aftermath from the perspective of the overall network, a coexpression network analysis with WGCNA was applied (Fig. 3a). All the DEGs at different stages were used to construct the coexpression network. Genes with similar expression patterns were classified into the same module, with colors used to distinguish among different modules (Fig. 3b). As shown in the hierarchical cluster tree in Fig. 3a, 10 different coexpression modules were identified by WGCNA. Correlation analysis demonstrated that these modules corresponded to a germination or post-germination stage-specific distribution pattern (Fig. 3c). Compared with other modules, for example, genes in the green module had the highest correlation with stage 3 (*p* = 4 × 10^− 8^, *r* = 0.92), whereas genes in the brown module displayed the highest correlation with stage 5 (*p* = 3 × 10^− 4^, *r* = 0.75). Genes in the pink module were relatively highly correlated with stages 1, 2, and 3 (Fig. 3c).

The module eigengene (ME), the first principal component of a gene module, represents the gene expression profile of each module except for the gray module (Miscellaneous). The eigengene expression profiles of eight groups of modules are shown in Fig. 3d. The ME of the blue module had higher expression levels during stages 1 and 2. MEs of green, pink, and red modules were expressed at higher levels during stage 3, while those of brown and magenta modules had higher expression levels during stages 4 and 5. The MEs of purple and turquoise modules had their highest expressions during stage 6.

To understand changes in biological processes in different modules, Fisher’s exact test was applied to identify enriched pathway categories and their mobilization trends in each module. The results of this test are given in Additional file 5: Table S5. The mobilization trend of enriched categories in different colored modules was obtained by comparing the number of background genes. Genes in the yellow module were associated with significant enrichment of seven categories: major CHO metabolism, glycolysis, RNA regulation of transcription, protein degradation, signaling, development, and cells. The blue module was significantly correlated with stages 1 and 2, with 12 significantly enriched categories identified (Additional file 5: Table S5). The number of enriched categories varied among the other modules. The smallest number of significantly enriched pathways, one each, was found in magenta and purple modules in the categories of signaling and RNA regulation of transcription, respectively.

**Fig. 4:**
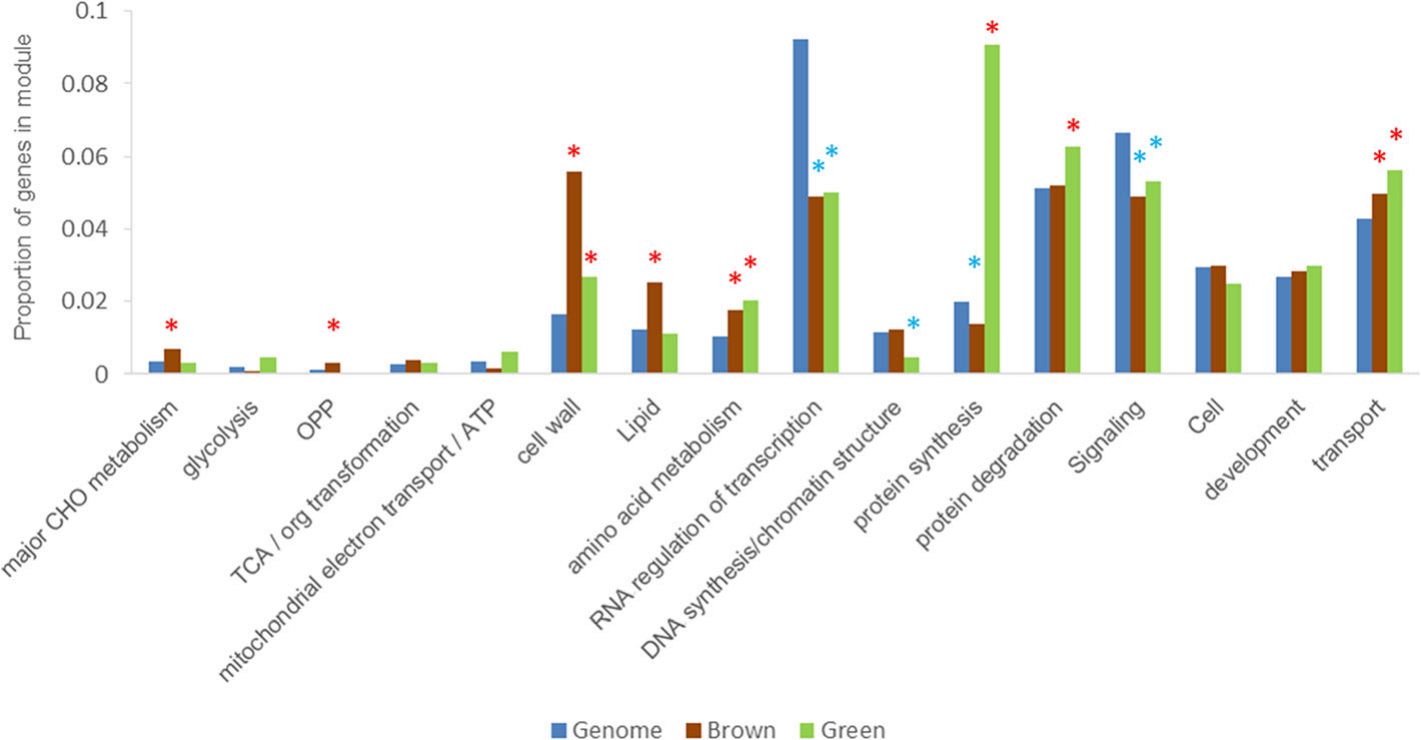
Enrichment pattern of different pathway categories in brown and green modules. The frequency of transcripts in each pathway category was calculated as a percentage of the cluster and compared with the percentage of the genome. Overrepresented and underrepresented functional groups in each cluster, as determined by Fisher’s exact test (*p* ≤ 0.05), are indicated with red and blue asterisks, respectively. For a complete list of all functional groups in all clusters, see Additional file 5: Table S5

Among the 10 modules, the green module was most strongly associated with stage 3, while the brown module was most relevant to stage 5 (Fig. 3c). The main analysis was thus performed on these two modules. Protein synthesis and degradation pathways were more active in the green module, whereas protein synthesis-related pathways were inhibited in the brown module. In the green module, the number of protein degradation pathway genes was elevated, but the increase was not significant (Fig. 4). The participation of protein synthesis and degradation pathways thus changed with different stages of germination. Surprisingly, the activity of the TCA cycle pathway was not significantly different among modules, consistent with the results shown in Fig. 2 and implying that the activity of this pathway is stable throughout seed germination and post germination.

**Fig. 5:**
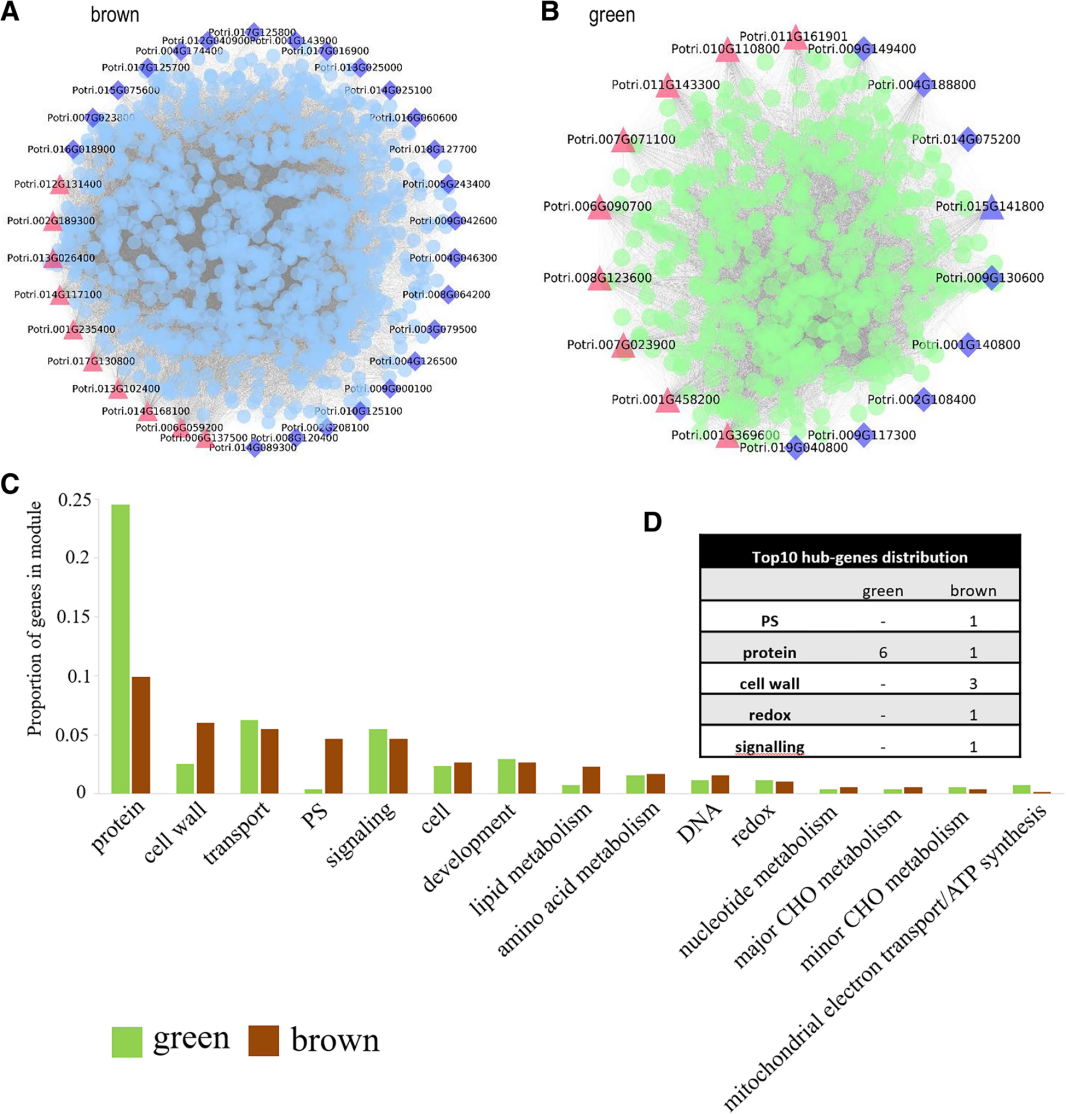
Brown- and green-module genes and networks. **a** Correlation network of genes in the brown module (stage 5). **b** Correlation network of genes in the green module (stage 3). Red triangles indicate the top-10 hub genes, and blue diamonds indicate transcription factors. **c** Distribution statistics of high-connectivity-node functional genes in brown and green modules. **d** Distribution of top-10 hub genes. A dash indicates the absence of hub genes associated with a given function in that module

WGCNA can also be used to construct gene networks in which each node represents a gene and the connecting lines between nodes, called edges, represent coexpression-related genes. The node gene with the highest connectivity, the hub gene, may play an important role in different modules. Brown and green module networks are shown in Fig. 5a and b, respectively. Some of the more interconnected node genes were those of transcription factors. For example, potri.015 g141800, the transcription factor gene with the highest connectivity in the green module, encodes *ANL2* (ANTHOCYANINLESS 2), a homeodomain protein belonging to the HD-GLABRA2 group that is highly similar to Arabidopsis AT4G00730 sequences. In a recent study, this gene was thought to be involved in the regulation of the root development process in Arabidopsis [38, 39], and its expression has been found to be significantly reduced in somatic embryo germ-defective plants of Norway spruce [40]. *ANL2* may therefore play a development-related regulatory role in poplar seed germination. The potri.016 g060600 gene, named *ZFP8* (ZINC FINGER PROTEIN 8), encodes a nuclear C2H2 zinc-finger protein and was found to have high connectivity within the brown module. In Arabidopsis, the protein encoded by *ZFP8* is a negative regulator of gibberellin-inducible seed germination and plays a key role in germination and seedling development [41]. Some of the less interconnected transcription factors, such as a *WUSCHEL-related homeobox 11* transcription factor (potri.019 g040800) found in the green module, are thought to be related to the germination of rice seeds [25]. In addition, a *YABBY* family member (potri.009 g000100) was located in the brown module; this transcription factor may play a key role in the germination of soybean seeds [42]. All of the above data are detailed in Additional file 6: Table S6.

Some highly connected functional genes were also found in different modules. The distribution statistics of high-connectivity-node functional genes in brown and green modules are given in Fig. 5c. In the green module, highly connected node genes associated with proteins, transport, signaling, and mitochondrial electron transport/ATP synthesis predominated, whereas more of such genes in the brown module were related to PS, cell walls, and lipid metabolism. One noteworthy issue is that these high-connectivity genes are not involved in key primary metabolic pathways such as glycolysis, TCA, and OPP.

To understand the types of highly connected genes in different modules, we analyzed the top 10 node genes with the highest connectivity distribution (Fig. 5d). In the green module, all high-connectivity genes belonged to protein categories, whereas functions of those in the brown module were distributed into the categories of PS, proteins, cell walls, redox, and signaling. All functional gene IDs and connectivities are listed in Additional file 7: Table S7.

### Primary metabolite analysis

**Fig. 6:**
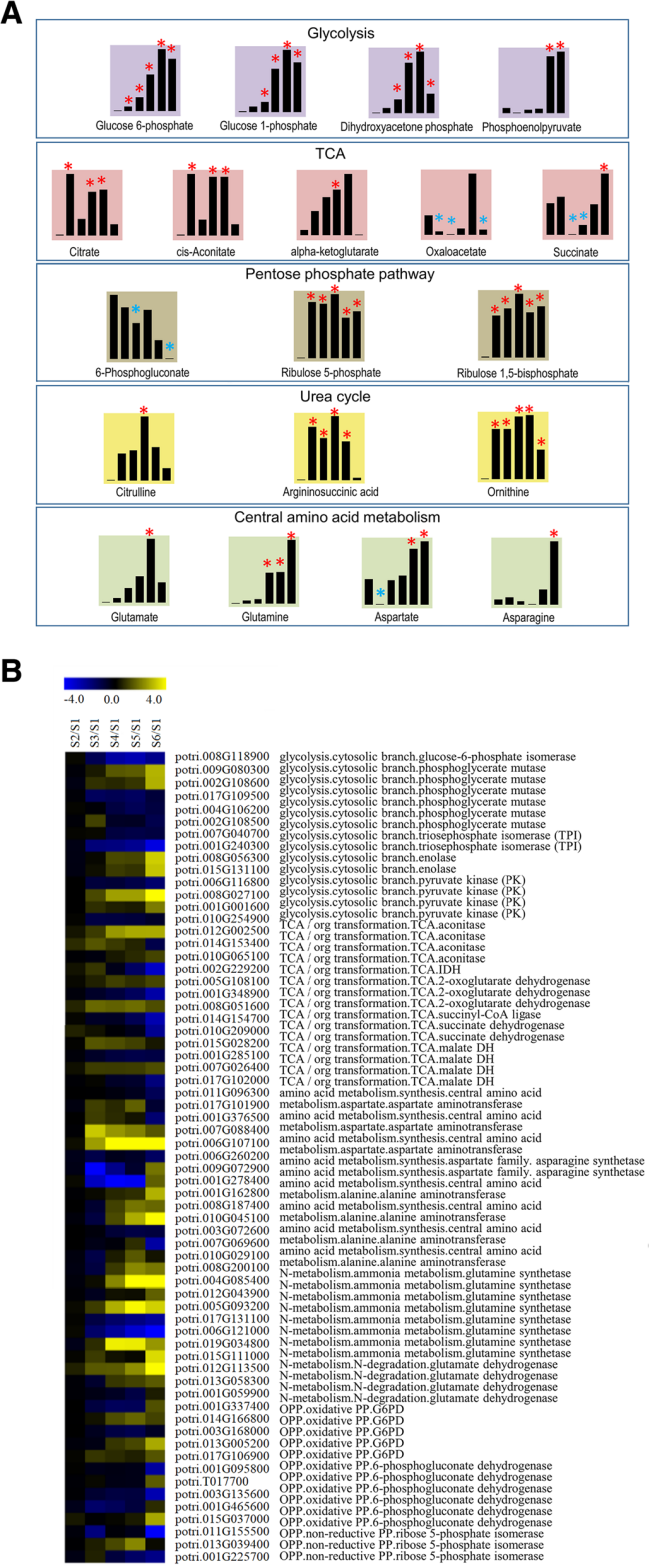
Profiling of primary metabolites and genes related to seed germination and post-germination processes. **a** Primary metabolite profiling of seed germination and post-germination processes. Asterisks indicate significant differences (red: significantly up-regulated; blue: significantly down-regulated). **b** Primary metabolism-related gene profiling of seed germination and post-germination processes. Expressions: blue, minimum; yellow, maximum. See also Additional file 8: Table S8 and Additional file 9: Table S9

In this study, an untargeted metabolomics assay was performed to detect metabolic changes at different stages of seed germination and post germination. Because glycolysis, TCA, and pentose phosphate pathways play crucial roles in plant primary metabolism, changes in these three pathways during poplar seed germination were studied using metabolomics. Given the importance of the urea cycle and central amino acid metabolism, metabolites of those two pathways were also considered. A total of 19 metabolites related to the above five pathways were identified. These metabolites experienced significant changes in content during at least one stage. All metabolites and their patterns of changes are shown in Fig. 6a. Four metabolites in the glycolysis pathway were identified. Among them, glucose 1-phosphate and glucose 6-phosphate are two main components of the hexose phosphate pool; the former is the decomposition product of starch, while the latter is closely related to glycolysis and pentose phosphate pathways. Glucose 6-phosphate was significantly increased at stage 2, while glucose 1-phosphate was significantly increased at stage 3. Both had their highest contents at stage 5. The other two substances were dihydroxyacetone phosphate and phosphoenolpyruvate. Dihydroxyacetone phosphate was significantly increased during stages 3 to 6, while phosphoenolpyruvate was only significantly increased at stages 5 and 6. Noteworthily, the patterns of changes in the contents of these four metabolites were extremely similar, as their contents increased significantly during the later stages of seed germination (Fig. 6a and Additional file 8: Table S8).

Five metabolites were closely related to the TCA: citrate, *cis*-aconitate, alpha-ketoglutarate, succinate, and oxaloacetate. Changes in the contents of citrate and *cis*-aconitate followed similar trends, i.e., significant increases at stages 2, 4, and 5, whereas levels of alpha-ketoglutarate increased during stages 1 to 5 but were only significantly increased at stage 4. Oxaloacetate underwent significant decreases at stages 2, 3, and 6, while succinate was significantly decreased at stages 3 and 4 and significantly increased at stage 6. In regard to metabolites related to the urea cycle, argininosuccinic acid exhibited a significant increase from stages 2 to 5, ornithine was significantly increased during stages 2 to 6, and citrulline was only increased significantly at stage 4. Two metabolites in the pentose phosphate pathway, ribulose 5-phosphate and ribulose 1,5-bisphosphate, exhibited a consistent pattern of change, with significant increases from stages 2 to 6. In the central amino acid pathway, glutamate significant increased at stage 5, and glutamine significantly increased from stages 4 to 6. Aspartate was significantly increased at stages 5 and 6, while asparagine was significantly increased at stage 6.

### Data integration analysis

To investigate the characteristics of primary metabolic processes during seed germination, transcriptome and metabolome data for the entire germination process were subjected to an integration analysis. Figure 6a, b and Additional file 8: Table S8 and Additional file 9: Table S9 show changes in primary metabolites and the expression patterns of related genes. We found that many genes encoding the same enzyme exhibited different expression patterns. For example, both potri.015 g131100 and potri.006 g116800 genes encode enolase but had opposite expression patterns, and the change in their target metabolite, phosphoenolpyruvate, was not consistent with either expression pattern. A similar phenomenon was observed for metabolic pathways such as the TCA cycle. These results indicate that the regulation of primary metabolism during poplar seed germination is very complicated; one possible reason is that the modulation of enzymatic activity in the primary metabolic pathways happens post-transcriptionally.

**Fig. 7:**
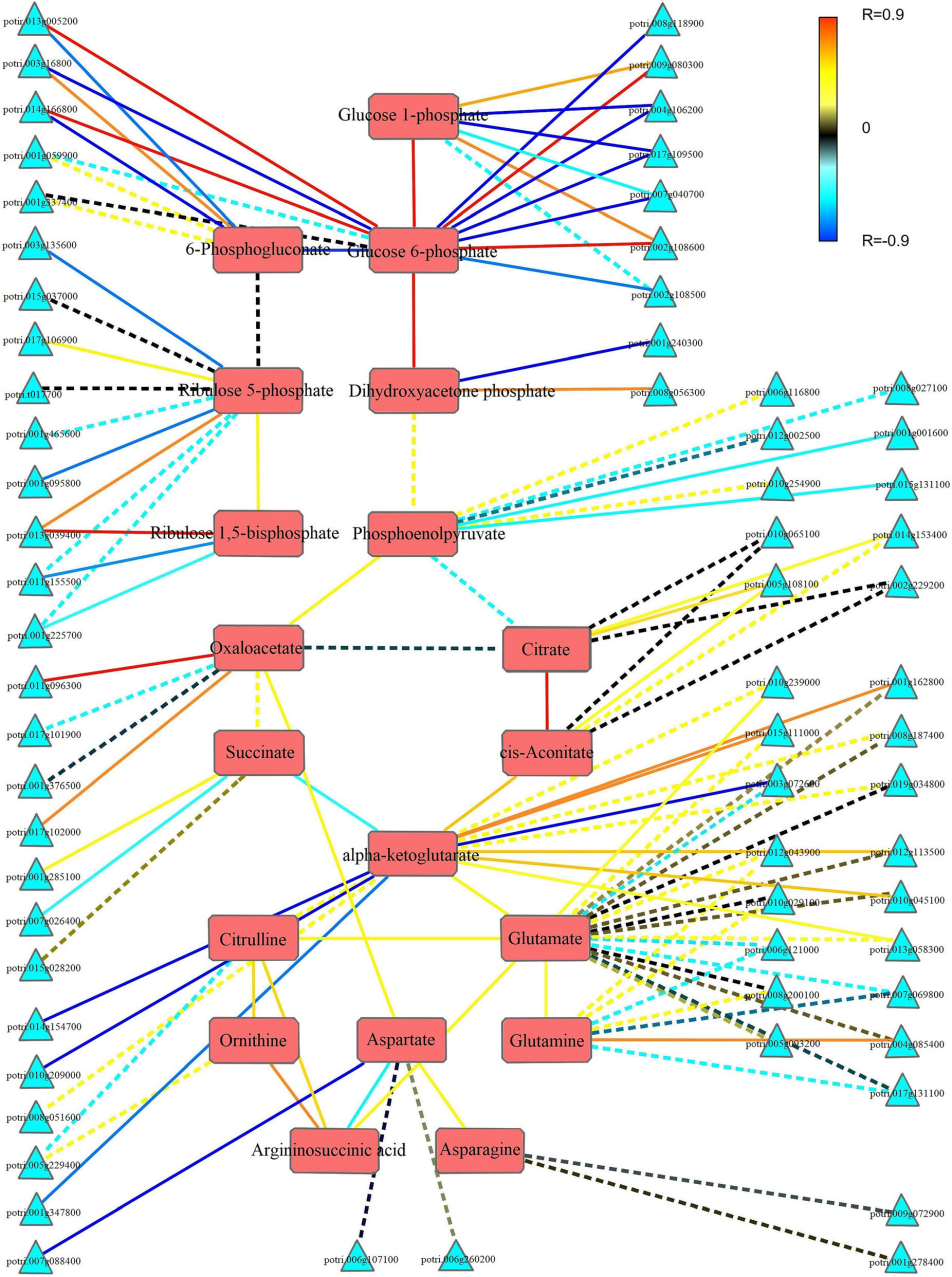
Targeted correlation network analysis (TCNA) between RNA-Seq and metabolomic data based on MapMan. Triangular and rectangular nodes indicate functional genes and metabolomic components, respectively. Significant and non-significant correlations (*p* ≤ 0.05) are represented by solid and dashed lines, respectively. Red line segments indicate positive correlations; blue lines show negative correlations

To reveal the interrelationships of genes and their associated metabolites, TCNA was applied to intrinsically link RNA-Seq and metabolomic data. Detailed results of this analysis are shown in Fig. 7 and Additional file 10: Table S10. Through this targeted correlation analysis, family members most strongly correlated to metabolites were identified. For example, phosphoenolpyruvate, an intermediate metabolite in the glycolytic pathway, is the product of enolase and is catalyzed by pyruvate kinase to yield pyruvate. Analysis of the correlation between the expression pattern of gene family members encoding the above-mentioned enzymes and the change trend of phosphoenolpyruvate revealed that potri.010 g254900 and potri.006 g116800 were highly correlated with phosphoenolpyruvate. Because pyruvate kinase, potri.010 g254900, is more closely related to phosphoenolpyruvate, this gene may play a key role in the pattern of phosphoenolpyruvate changes.

A strong positive correlation was uncovered between the change patterns of three metabolites: alpha-ketoglutarate, glutamate, and glutamine. The two families of genes controlling the synthesis and catabolism of alpha-ketoglutarate were negatively correlated with all isocitrate dehydrogenase and alpha-ketoglutarate dehydrogenase genes except for potri.008 g051600 (encoding alpha-ketoglutarate dehydrogenation). In the glutamate synthesis pathway, genes encoding alanine aminotransferase and glutamate dehydrogenase generally were strongly positively correlated with alpha-ketoglutarate and glutamate. Genes encoding glutamine synthetase typically have features highly associated with glutamate and glutamine.

Adjacent metabolites in metabolic pathways generally have strongly related characteristics, and this correlation is typically positively correlated. As shown in Fig. 7, a positive correlation trend was found among most contiguous metabolites, which suggests that metabolite changes under the same pathway are generally consistent. In contrast, significant differences were found between glucose 6-phosphate and 6-phosphogluconate, 6-phosphogluconate and ribulose 5-phosphate, phosphoenolpyruvate and citric acid, and alpha-ketoglutarate and succinic acid, with a significant negative correlation observed between changes in the contents of these metabolites.

## Discussion

Poplar is a model woody plant widely used in basic research. Because asexual reproduction is prevalent in poplar, the molecular mechanism of seed germination and its aftermath in poplar has not been investigated. In this study, samples from dry seed to seedling stages were collected and used for RNA-Seq and metabolomic analyses. Two different bioinformatic methods, *K*-means and WGCNA, were used to analyze the RNA-Seq data. This analysis produced several novel insights, including the identification of a series of time-specific modules, hub genes and transcription factors, some of the latter of which were consistent with previous research results. Many stress, cell wall, and protein-related genes were found to have a close relationship with germination and post-germination stages, while changes in glycolysis, oxidative phosphorylation, and the TCA were not significant. Further analysis showed that some metabolites in these pathways undergo significant changes at different stages of germination and that metabolite change patterns have similar characteristics under the same metabolic pathway. Enzymes associated with changes in metabolite content are commonly encoded by multiple gene families, and some members of these families were highly correlated with metabolite changes. Our study identified important genes and metabolites associated with germination and post-germination processes of poplar seeds and has laid a foundation for future biochemical and functional studies.

The seed germination process of *Arabidopsis thaliana*, rice, and soybean has been extensively studied. For example, two transcription factor genes, *ZFP8* and *WUSCHEL-related homeobox 11*, as well as a *YABBY* family member are considered to be closely related to seed germination [25, 41, 42]. Howell et al. (2009) found that the transcriptional abundance of sugar metabolism-related genes increased significantly in the late stage of rice seed germination [25]. Studies on the germination of barley seeds have shown that genes involved in amino acid metabolism are expressed at 48 h after imbibition [43]; this observation is consistent with the results shown in Fig. 4, which suggests that germination is a highly conserved process among higher plants. In contrast, however, we found that transcription factors such as *CPP (Zn)* and *DOF* are specifically expressed at different stages of seed germination, a phenomenon not observed in previous studies. We speculate that this discrepancy is due to differences among studied species.

Genes in green and brown modules in the WGCNA were found to be most relevant to stages 3 (slow water absorption period) and 5 (cotyledon expansion period), respectively. High-connectivity-node functional genes in green modules were associated with protein synthesis and decomposition, whereas those in brown modules were associated with CHO, cell wall, lipid metabolism and PS. We speculate that changes first occur in the early protein metabolism pathway, with significant changes in saccharide metabolism taking place during the cotyledon expansion period. Because the WGCNA method does not allow discovery of gene modules most closely related to other periods, such as rapid water absorption or hypocotyl elongation, we were unable to detect events occurring at the transcriptional level at each stage of seed germination. We plan to explore this issue in future research.

In this study, we used two different bioinformatics algorithms, *K*-means and WGCNA, to analyze transcriptome data, a strategy similar to that applied in previous studies of strawberry and *Brassica oleracea* [44, 45]. Although these two methods provided us with different options for finding important biological events occurring at different seed germination and post-germination stages, both suggested that glycolysis and the TCA cycle were in a steady state at the transcriptional level. This observation is inconsistent with previous findings [22, 25, 43, 46–51] and does not explain the changes in the metabolic levels of these two pathways. We thus needed to determine the causes of these metabolite changes and to identify the genes most likely closely related. Using the TCNA method, we were able to identify gene family members most closely related to changes in the content of specific metabolites. These results provide us with possible directions for subsequent determination of genes to be studied, and this combined method is also potentially useful for future multiomics joint analyses.

Correlation analysis of metabolites during the germination process revealed a significant positive correlation among the most relevant metabolites. A significant difference was found, however, between glucose 6-phosphate and 6-phosphogluconate, 6-phosphogluconate and ribulose 5-phosphate, phosphoenolpyruvate and citric acid, and alpha-ketoglutarate and succinic acid. These transformations are situated at junctions with other metabolic pathways, and a strong correlation exists between branch metabolites and upstream metabolites. We have therefore inferred that substances at these branch sites flow in multiple, major directions. These results can thus serve as a useful reference for further study of metabolite flow.

At the transcriptional level, many genes undergo complex changes during germination, even though the expression patterns of genes encoding the same enzyme are dissimilar. At the metabolic level, however, only a few significant changes to metabolites were observed (Fig. 6a, b). This result is probably due to the fact that a significant number of low-abundance metabolites cannot be detected by HPLC-MS/MS relative to RNA-Seq. Another possibility is that the level of transcription is a complex process: just a few genes may play a key role in a specific germination stage; in addition, post-transcriptional regulation may act as a buffer for this process, leading to very few changes at metabolic levels [52].

## Conclusions

In this study, we investigated poplar seed germination and post-germination biological processes using a transcriptional combinatorial metabolomic method. In contrast to time-sequential methods used previously, we relied on a strategy based on morphological differences, which can better address the combination of molecular level and morphology. We compared our results with those obtained in earlier investigations of the germination of Arabidopsis [21, 53], rice [25], and barley [20, 43] and found that seed germination is a highly conserved process among higher plants. Using a combination of *K*-means and WGCNA methods, we identified transcription factors and metabolic pathways playing an important role in specific stages of seed germination. The application of targeted network analysis methods allowed us to discover candidate genes participating in specific processes. Such a method also represents a possible strategy for future joint sequential multiomics analyses. Our study was the first to use an integrated approach in regard to seed germination in poplar. The results of our study are thus an important starting point for future analyses and provide new insights to explore alternative strategies and possibilities.

## Methods

### Plant materials, experimental conditions, and fresh weight measurements

Seeds of poplar (*Poplar simonii × Poplar nigra*) were collected from the same female poplar plants producing half-sib families at the Experimental Forest Farm of Northeast Forestry University (Harbin, China). Germination assays were carried out on three replicates (50 seeds per replicate). Seeds were incubated in covered plastic boxes at 25 °C on two sheets of absorbent paper moistened with 4.5 mL of distilled water. After removing excess moisture with absorbent paper, 20 germinating seeds were weighed (to ±0.0001 g) every 15 min at an ambient temperature of 25 ± 1 °C. Transcriptomic and metabolic profiling was performed at specific time points based on the fresh weights of germinated seeds. After weighing, seeds were immediately blotted with absorbent paper, snap frozen in liquid nitrogen, and subjected to transcriptomic and metabolic analyses.

### Transcriptome profiling and analysis

Total RNA was extracted from approximately 200 seeds using Trizol reagent (Invitrogen, Carlsbad, CA, USA) and then incubated with 10 U DNase I (Takara, Dalian, China) for 30 min at 37 °C to remove genomic DNA. Three experimental replicates per sample were used for RNA library construction. RNA-Seq libraries were prepared according to the Illumina Library Prep Kit manufacturer’s protocol. After sequencing, raw data were filtered to remove adaptor contamination and low-quality reads. All clean reads were then mapped to the poplar genome, which was downloaded from the Phytozome website (https://phytozome.jgi.doe.gov/pz/portal.html), using the HISAT2 alignment algorithm with default parameters [54]. Mapped reads of each gene were extracted using SAMtools [55]. DEGs were identified in NOISeq using fold change ≥2 and *p* ≥ 0.8 thresholds [56]. Gene expression levels were normalized using the FPKM method.

### Clustering of gene expression data

*K*-means clustering with Euclidean distances in MeV4.8 [57] yielded 24 clusters based on input FPKM values. Coexpression networks were constructed using the WGCNA package in R [58]. Eigengene values were calculated for each module and used to test associations with each germination stage. Subsequent analyses were performed using *r* ≥ 0.75 and *p* ≤ 0.05 thresholds (Student’s *t*-test). Networks were visualized using Cytoscape v.3.5.1 [59].

### Enrichment analysis

The MapMan tool was used to assign pathway categories and screen transcription factors [37]. Fisher’s exact test was used to identify categories significantly enriched in DEGs (*p* ≤ 0.05 threshold).

### Metabolomic analysis

Analysis of metabolites was performed using 50 mg of poplar seed collected from different germination periods, LC-MS/MS analyses were carried out on an UHPLC system (1290, Agilent Technologies, USA) equipped with a UPLC BEH Amide column (1.7 μm, 2.1 × 100 mm, Waters) coupled to a TripleTOF 6600 system (Q-TOF, AB Sciex) operating in EI mode. The mobile phase (A) consisted of 25 mM NH_4_OAc and 25 mM aqueous NH_4_OH (pH = 9.75). Gradient elution was carried out at a delivery rate of 0.3 mL min^− 1^ using the mobile phase (A) and acetonitrile (B) as follows: 0 min, 85% B; 2 min, 75% B; 9 min, 0% B; 14 min, 0% B; 15 min, 85% B; and 20 min, 85% B. The injection volume was 2 μL. Six biological repeats were performed, with all other details as described in a previous study [60]. During data processing, two steps were used to determine significant differences in metabolites at different times. In the first step, first principal component of variable importance in the projection (VIP) values were obtained. Changed metabolites were considered to be those with VIP values exceeding 1.0. In the second step, the remaining variables were assessed by Student’s *t*-test (*p* > 0.05), and variables were discarded between the two comparison groups [61].

### TCNA

TCNA is used to evaluate the contribution of each multigene family member to the change in a target metabolite. In this method, gene family members encoding proteins directly involved in the “synthesis” and “decomposition” of a target metabolite are considered. Genes responsible for target metabolite synthesis and decomposition were identified on the basis of gene annotation and metabolite rankings obtained from annotation information generated with the MapMan tool. A Spearman correlation analysis was performed and visualization was carried out using Cytoscape software (3.5.1) based on *p* ≥ 0.05 (Student’s *t*-test) and r ≥ 0.25 thresholds.

## Additional files

Additional file 1: **Table S1.** Changes in fresh weight of poplar seeds during germination. (XLSX 15 kb)

Additional file 2: **Table S2.** The average number of raw reads per library and matching results. (XLSX 17 kb)

Additional file 3: **Table S3.** IDs of genes in the 24 clusters. (XLSX 162 kb)

Additional file 4: **Table S4.** The relative abundance of transcription factors at different stages. (XLSX 17 kb)

Additional file 5: **Table S5.** Enriched pathway categories and mobilization trends in each module. (XLSX 22 kb)

Additional file 6: **Table S6.** High-connectivity transcription factors. (XLSX 16 kb)

Additional file 7: **Table S7.** Brown module and green module functional gene IDs and connectivity. (XLSX 48 kb)

Additional file 8: **Table S8.** Primary metabolite changes during seed germination and post germination. (XLSX 10 kb)

Additional file 9: **Table S9.** Primary metabolism-related gene profiling. (XLSX 22 kb)

Additional file 10: **Table S10.** Genes and correlations involved in a targeted correlation network analysis (TCNA). (XLSX 14 kb)

## Data Availability

All datasets generated or analyzed during this study are available from the corresponding author upon reasonable request.

## Abbreviations

AP2/EREBP: APETALA2/ethylene-responsive element binding proteins
CPP (Zn): Cystein rich polycomb like protein (Zn)
DEGs: Differentially expressed genes
DOF: DNA binding with one finger
EIL: Ethylene-insensitive3 -like
HPLC-MS/MS: High-performance liquid chromatography-mass spectrometry/mass spectrometry
ME: Module eigengene
TCA: Tricarboxylic acid cycle
TCNA: Targeted correlation network analysis
VIP: Variable importance in the projection
WGCNA: Weighted gene coexpression network analysis
